# Histopathological, Demographic, and Clinical Signatures of Medulla Oblongata Germ Cell Tumors: A Case Report With the Review of Literature

**DOI:** 10.7759/cureus.51861

**Published:** 2024-01-08

**Authors:** Daisuke Sato, Shota Tanaka, Hirokazu Takami, Shunsaku Takayanagi, Yurie Rai, Munetoshi Hinata, Atsuto Katano, Nobuhito Saito

**Affiliations:** 1 Department of Neurosurgery, The University of Tokyo Hospital, Tokyo, JPN; 2 Department of Pathology and Diagnostic Pathology, The University of Tokyo Hospital, Tokyo, JPN; 3 Department of Radiology, The University of Tokyo Hospital, Tokyo, JPN

**Keywords:** brainstem tumor, medulla oblongata, hydrocephalus, germ-cell tumor, germinoma

## Abstract

The medulla oblongata is one of the rarest sites of occurrence for germ cell tumors (GCTs) of the central nervous system. As there is scant data regarding epidemiology, clinical presentations, optimal intervention, and long-term prognosis, we aimed to delineate the features of this rare entity by presenting our representative case and performing a quantitative review of the literature. A 24-year-old woman presented to our department with vertigo and swallowing difficulties. Magnetic resonance imaging revealed a homogenously enhanced exophytic lesion arising from the medulla oblongata and extending to the fourth ventricle. Surgical resection was performed and a histological diagnosis of pure germinoma was made. The patient underwent chemotherapy and whole-ventricular irradiation. No recurrence has been experienced for 4 months after the surgery. According to the literature, the prognosis of GCTs at the medulla oblongata seems no worse than those at typical sites. Striking features including occurrence at an older age, female preponderance, and a predominance of germinoma were noteworthy. The pattern of local recurrence suggests extensive radiation coverage is not a prerequisite. Special attention is needed for cardiac and respiratory functions as the main factors eliciting mortality.

## Introduction

Germ cell tumors (GCTs) cover a spectrum of neoplastic diseases derived from primordial germ cells at different stages of maturation [1-3]. GCTs are classified based on the distinct developmental potency of the primordial germ cells [1]. Although the gonads are the dominant location, 1-5% of GCTs arise in extragonadal sites [4]. During the early stage of embryogenesis, primordial germ cells migrate from the yolk sac wall towards the genital ridge, consequently forming the gonads. Primordial germ cells that have topographically mis-migrated and circumvented apoptosis or elimination by the immune system are presumed to lead to extragonadal GCTs [1,2,5]. The anatomical distribution of extragonadal GCTs along the midline of the body reflects the migration route of primordial germ cells [1,6]. Such cells are typically implanted in the midline of the sacrum, retroperitoneum, mediastinum, and midline of the central nervous system (CNS) [7]. Typical locations in the CNS are the pineal gland, followed by the neurohypophysis and lateral/third ventricles, accounting for 80-90% [8-11]. Atypical locations include the basal ganglia and thalamus, among others [12-14], and occurrence at the medulla oblongata is rare.

CNS GCTs have been known to demonstrate site-specific clinical and histopathological presentations. Sex is tightly linked to the site of occurrence, with cases at the neurohypophysis generally showing equality between sexes [15], while pineal gland GCTs predominantly occur in males (approximately 90%) [16]. Histopathology is also site-dependent, as germinoma predominates at the neurohypophysis, while non-germinomatous GCTs (NGGCTs) commonly arise at the pineal gland [8]. Little is known about the clinical and histopathological presentations for GCTs at atypical sites, much less for medulla oblongata GCTs, particularly in terms of clinical behaviors. While chemotherapy (CMT) regimens and radiation fields have been historically investigated for GCTs at typical sites in multiple clinical trials worldwide [10,17-24], the most appropriate treatment strategies, such as optimal radiation field based on relapse patterns, remain poorly characterized [25]. Germinomas at atypical sites outside of these midline structures have been known to show worse prognoses than those at typical sites [8].

This study examined the literature for clinical and histopathological findings of GCTs arising at the medulla oblongata [26-54]. This study aimed to elucidate the characteristic features of this atypical occurrence compared to those of GCTs arising from typical locations.

## Case presentation

**Figure 1:**
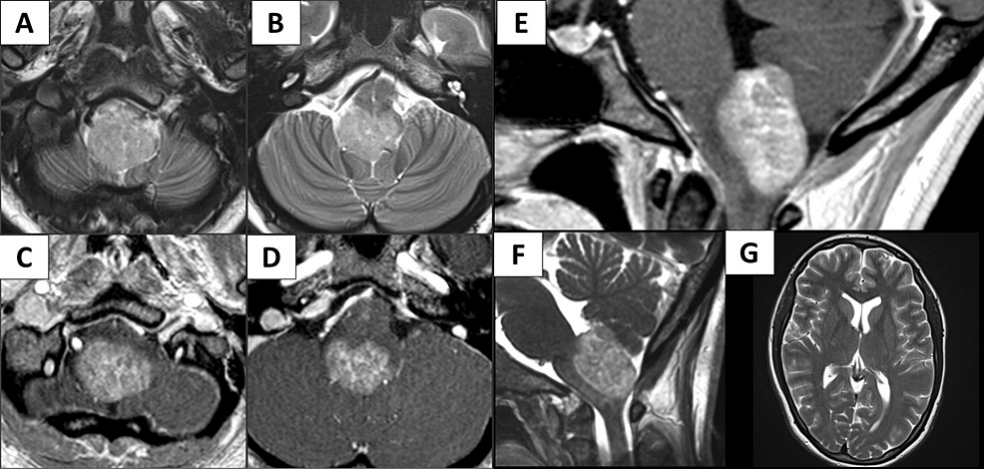
Pre-operative images of the representative case Magnetic resonance imaging revealed a solid mass occupying the medulla oblongata (A). The lesion exhibited T2 high intensity and was compressing the surrounding structures (B). Homogenous gadolinium enhancement was observed (C, D). The lesion was located at the dorsal aspect of the medulla oblongata and was protruding to the fourth ventricle (E, F). Hydrocephalus was not evident (G).

A 24-year-old woman without any contributory medical history was referred to our department with progressively worsening vertigo and mild swallowing difficulties over the past few weeks. A neurological examination revealed an unstable gait. Ocular movements were intact and congruent. No ataxia or cranial nerve palsy was evident, except for mild impairment of deglutition. Magnetic resonance imaging (MRI) revealed a homogenously enhancing exophytic lesion arising from the medulla oblongata and extending to the fourth ventricle (Figure 1A-1D). The lesion was spreading through the roof of the fourth ventricle, sparing the floor of the fourth ventricle, vermis, and tonsil (Figure 1E-1F). Hydrocephalus was not present (Figure 1G). CSF cytology was normal (class I). Levels of tumor markers (alpha-fetoprotein, beta-human chorionic gonadotropin) showed no abnormalities. No spinal cord lesion was observed on MRI. No other systemic lesion was present on computed tomography or positron emission tomography, reducing the likelihood of metastasis. Differential diagnoses included pilocytic astrocytoma, posterior fossa ependymoma, medulloblastoma, and diffuse midline glioma, H3 K27-altered. 

**Figure 2:**
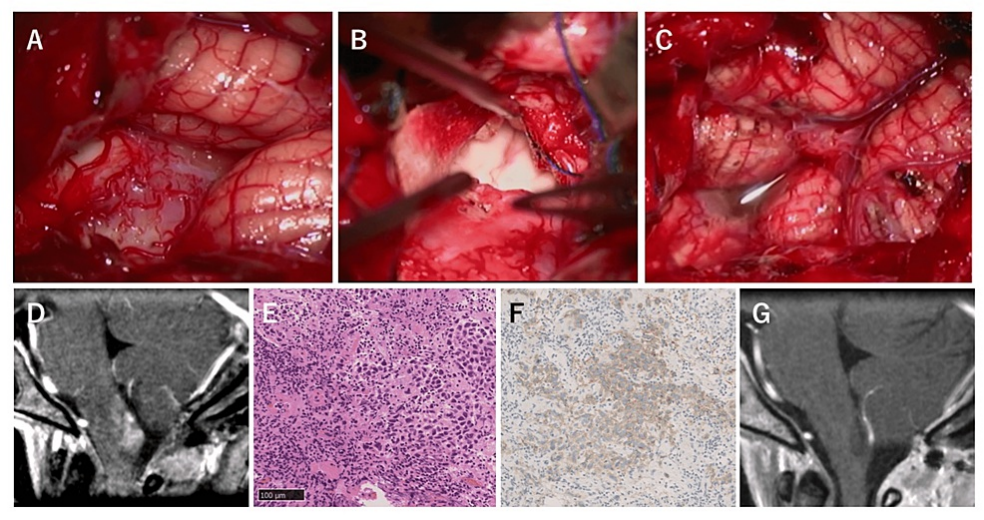
Intraoperative findings and postoperative images of the representative case The lesion was pinkish and was surrounded by engorged tortuous vessels (A). The lesion extended along the tela choroidea, and the floor of the fourth ventricle was spared (B). Subtotal resection was performed without any damage to the surrounding vital structures (C). Postoperative imaging revealed sufficient tumor resection without unintentional brainstem damage (D). Histologically, the tumor was composed of small lymphocyte infiltration and pleomorphic tumor cells with large eosinophilic cytoplasm and irregularly shaped nucleus (E). C-kit was positive for the cytoplasm of the tumor cells (F). Follow-up imaging after chemoradiotherapy showed no tumor recurrence (G).

To alleviate the neurological symptoms and obtain a pathological diagnosis, surgical resection was proposed. The patient and her family consented to the treatment. Under general anesthesia, the lesion was approached via a midline suboccipital craniotomy and C1 laminectomy. The tumor arose from the medulla oblongata's dorsal aspect with an indistinguishable border and protrusion to the cerebellomedullary fissure (Figure 2A). Dissection of the cerebellomedullary fissure bilaterally revealed the extension of the tumor through the tela choroidea to the inferior medullary velum, while the foramen of Magendie was spared. Further, the inner surface of the cerebellum and the floor of the fourth ventricle were intact (Figure 2B). As intraoperative histopathological diagnosis was elusive, subtotal resection was performed (Figure 2C).

 

The patient quickly recovered from anesthesia and the surgery without any postoperative complications. Improvements in vertigo and deglutition were observed, with these symptoms vanishing a few days after the surgery. Postoperative imaging revealed a subtotal resection of the tumor, with no unintentional damage to the surrounding structures (Figure 2D). Histopathological exploration demonstrated abundant infiltration of lymphocytes and sheets of large atypical cells with large, irregularly shaped nuclei and clear cytoplasm, constituting the classical “two-cell pattern” (Figure 2E). Immunohistochemistry showed positive results for c-kit (Figure 2F), podoplanin, and placental alkaline phosphatase. A histopathological diagnosis of pure germinoma was made.

The patient underwent further treatment with CMT comprising carboplatin and etoposide, and intensity-modulated radiation therapy (IMRT) with a dose of 24 Gy to the whole ventricles. She has been followed up every two months, and no recurrence has been observed 4 months after the surgery (Figure 2G). Written informed consent was obtained from the patient.

## Discussion

Literature review

Methods

**Figure 3:**
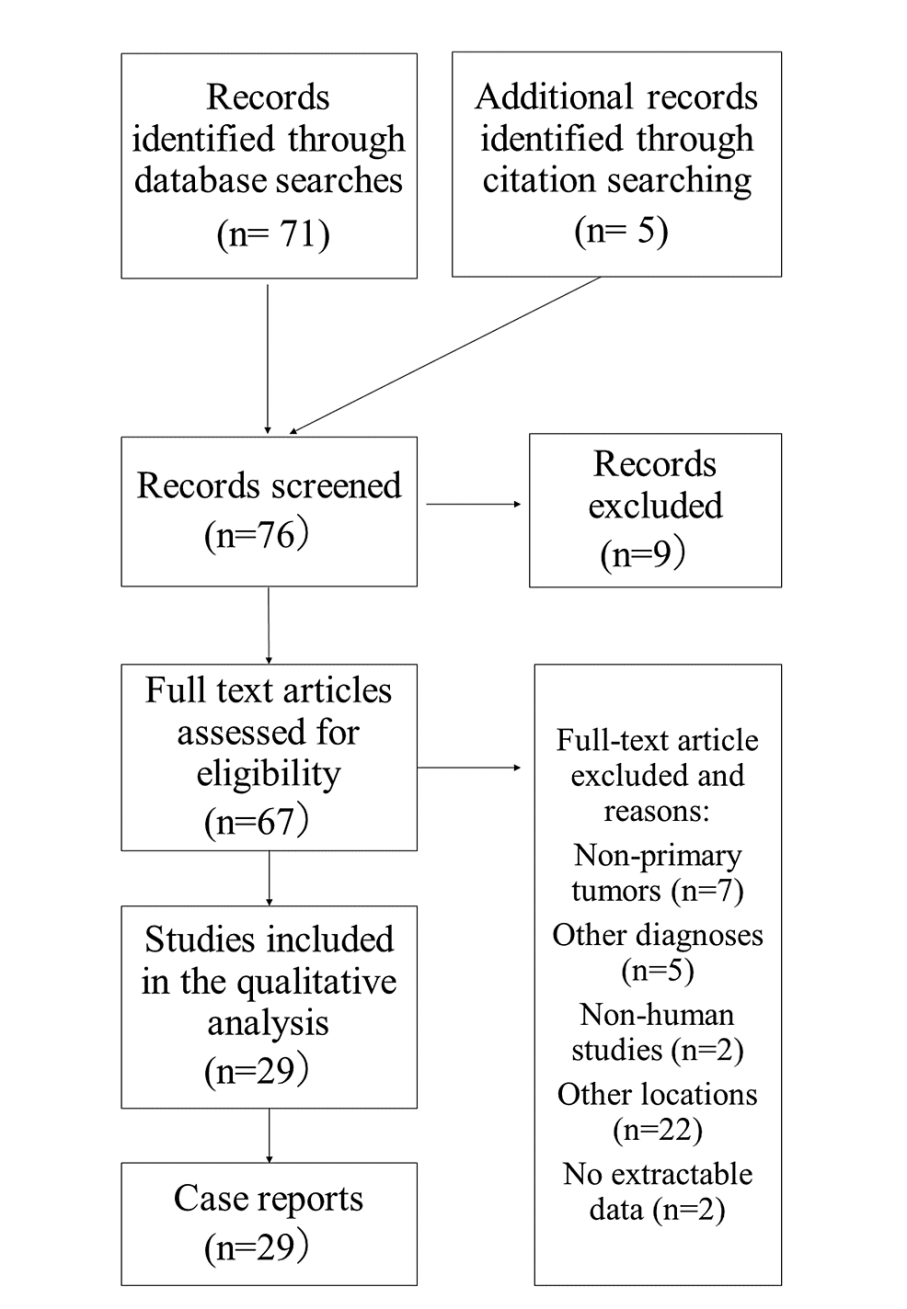
Flow chart of the study selection process The quantitative review retrieved literature published from January 1983 to December 2022 related to primary germ cell tumors (GCTs) involving the medulla oblongata and/or fourth ventricle. A total of 29 studies describing 33 cases were identified. We added our case to this cohort.

A quantitative review of the literature was conducted in December 2022. PubMed was queried using search terms such as “germinoma or germ cell tumor (s) or teratoma” and “medulla or fourth ventricle”. An exhaustive search of citations was also conducted. Two authors (D.S. and H.T.) scrutinized the source and data. Inclusion criteria were all case reports and case series regarding primary (neither recurrent nor metastatic) GCT of the medulla oblongata. No large-scale studies have been reported due to the rarity of the disease. Reports describing irrelevant cases were excluded. Non-English language publications and reports with unclear clinical descriptions were also excluded. A flow chart of the study selection process is presented in Figure 3. 

Data Extraction

Data extraction was performed by two independent investigators (D.S. and H.T.). The information collected was as follows: age, sex, tumor location, evidence of hydrocephalus at initial presentation, findings from CSF cytology, treatment modalities (extent of surgical resection, field, and dose of radiotherapy {RT} and CMT regimens), presence of dissemination in the CSF space on imaging, and treatment outcomes (relapse and survival).

Statistical Analysis

Our case was added to this pooled data. All statistical analyses were carried out using JMP version 16 software (SAS Institute Inc., Cary, NC, USA).

Results of Review

The quantitative review retrieved literature published from January 1983 to December 2022 related to primary GCTs involving the medulla oblongata and/or fourth ventricle. A total of 29 studies describing 33 cases were identified. We added our case to this cohort.

The entire cohort (Supplementary Table 1) included 15 males (44%) and 19 females (56%), with a mean age of 22.2 years (range, 9-50 years; median, 22 years).

**Table 1:** Summary of the reported cases and our representative case Clinicopathological characteristics and details of the treatment are all summarized. BLM, bleomycin; CBCDA, carboplatin; CDDP, cisplatin; GTR, gross total resection; IFOS, ifosfamide; PR, partial resection; STR, subtotal resection; VP-16, etoposide

Author (year)	Age/Sex	Histological diagnosis	Germinoma component	Extent of resection	Hydrocephalus	Area of irradiation	Chemotherapy	Initial cytology	Concomitant dissemination	Degree of lymphoplasmacytic infiltration	Relapse	Relapse free period	Form of relapse	Follow-up period	Survival
Iwasaki et al., 1984 [26]	12y/F	Teratoma with a prominent rhabdomyogenic element and germinoma	Yes	GTR	Evident	Radiation (not otherwise described)	-	-	No	Moderate	No	18 days	-	18 days	Dead (Pneumonia and gastrointestinal bleeding)
Poungvarin et al., 1991 [27]	17y/M	Germinoma	Yes	PR	No	Radiation (not otherwise described)	-	-	No	Moderate	No	3 months	-	3 months	Dead (Pneumonia)
Hashimoto et al., 1992 [28]	19y/F	Germinoma	Yes	PR	No	Whole brain 30Gy + Tumor site 30Gy + Spinal cord 35Gy	-	-	No	Sparse	No	2 months	-	2 months	Alive
Tashiro et al., 1993 [29]	30y/F	Germinoma	Yes	PR	No	Tumor site 40Gy	1st: CDDP + VP-16; 2nd: CBCDA + VP-16	-	Suprasellar lesion	Moderate	Yes	10 months	Local recurrence	14 months	Alive
Sugiyama et al., 1994 [30]	32y/F	Germinoma	Yes	PR	No	Tumor site 44Gy + Spinal cord 20Gy	-	-	No	Moderate	No	9 years	-	9 years	Alive
Israel et al., 1996 [31]	17y/M	Choroid plexus papilloma with germinoma	Yes	STR	No	Whole ventricular system and tumor site (not otherwise described)	CDDP + VP-16 + BLM	No abnormality	No	Moderate	No	18 months	-	18 months	Alive
Nakajima et al., 2000 [32]	18y/F	Germinoma	Yes	PR	No	Gamma knife surgery	CBCDA + VP-16	-	No	Moderate	No	8 months	-	8 months	Alive
Tsuzuki et al., 2001 [33]	39y/M	Immature teratoma	No	PR	No	Radiation (not otherwise described)	Chemotherapy (not otherwise described)	-	No	Moderate	Yes	3 months	Local recurrence	3 months	Dead (Respiratory failure)
Yoshida et al., 2003 [34]	33y/M	Germinoma	Yes	STR	No	-	CBCDA + VP-16	No abnormality	No	Moderate	No	7 months	-	7 months	Alive
Yen et al., 2003 [35]	16y/F	Germinoma	Yes	STR	Mild	Whole brain 30 Gy + Tumor site 50Gy + Spinal cord 30Gy	-	-	No	Moderate	No	7 years	-	7 years	Alive
Kakani et al., 2006 [36]	16y/F	Germinoma	Yes	STR	No	-	-	No abnormality	Suprasellar lesion	Moderate	No	12 days	-	12 days	Dead (Cardiac arrest)
Yang et al., 2009 [37]	12y/M	Germinoma	Yes	STR	No	10 weeks (not otherwise described)	CDDP + IFOS	-	No	Moderate	No	6 months	-	6 months	Alive
Akimoto et al., 2009 [38]	30y/F	Germinoma	Yes	STR	No	Tumor site (not otherwise described)	Chemotherapy (not otherwise described)	-	No	Sparse	No	12 months	-	12 months	Alive
Akimoto et al., 2009 [38]	24y/M	Germinoma	Yes	STR	No	Tumor site (not otherwise described)	Chemotherapy (not otherwise described)	-	No	Sparse	No	8 months	-	8 months	Alive
Madden et al., 2009 [39]	12y/M	Germinoma	Yes	STR	No	Whole brain 21Gy + Tumor site 9Gy + Spinal cord 21Gy	CBCDA + VP-16	-	No	Moderate	No	12 months	-	12 months	Alive
Madden et al. , 2009 [39]	21y/M	Teratoma with germinoma and embryonal carcinoma elements	Yes	GTR	No	Posterior fossa 21.6Gy + Tumor site 32.4Gy	CBCDA + VP-16 + BLM	-	No	Moderate	Yes	2.5 years	Local recurrence	3.5 years	Dead (Respiratory failure)
Neelima et al., 2010 [40]	24y/F	Germinoma	Yes	GTR	No	-	-	-	No	Moderate	No		-	-	Alive
Yasuhara et al., 2011 [41]	27y/F	Germinoma	Yes	PR	No	Whole ventricular system 30Gy + Tumor site 20Gy	IFOS + CDDP + VP-16	-	No	Moderate	No	11 months	-	11 months	Alive
Shuto et al., 2012 [42]	28y/M	Germinoma	Yes	GTR	No	Tumor site 45Gy + Spinal cord 27Gy	CBCDA + VP-16	-	No	Moderate	No	3 years	-	3 years	Alive
Nakatsuka et al., 2012 [43]	31y/F	Germinoma	Yes	STR	No	Whole ventricular system 24Gy + Tumor site 24Gy	CBCDA + VP-16	-	No	Moderate	No	6 months	-	6 months	Alive
Hao et al., 2013 [44]	14y/M	Germinoma	Yes	STR	No	Gamma knife surgery	CDDP + VP-16 + BLM	-	No	Moderate	No	4 years	-	4 years	Alive
Hao et al., 2013 [44]	22y/F	Germinoma	Yes	STR	No	-	-	-	No	Moderate	Yes	7 months	Local recurrence	8 months	Dead (Pneumonia)
Khan et al., 2013 [45]	25y/F	Germinoma	Yes	PR	No	Whole brain 24Gy + Tumor site (posterior fossa and spinal cord) 16Gy + Spinal cord 24 Gy	-	-	Spinal cord	Dense	No	10 months	-	10 months	Alive
Li et al., 2014 [46]	9y/M	Mature teratoma	No	GTR	No	-	-	-	No	Slight	No	22 days	-	22 days	Dead (CNS infection)
Li et al., 2014 [46]	10y/M	Immature teratoma	No	GTR	No	-	IFOS + CDDP + VP-16	-	No	Sparse	No	59 months	-	59 months	Alive
Yip et al., 2014 [47]	22y/F	Germinoma	Yes	GTR	No	Whole ventricular system 30.6Gy + Tumor site 45Gy	-	-	No	Moderate	No	12 months	-	12 months	Alive
Budohoski et al., 2016 [48]	23y/F	Germinoma	Yes	GTR	No	Whole brain 25Gy + Tumor site 15Gy + Spinal cord 25Gy	-	-	No	Sparse	No	12 months	-	12 months	Alive
Seifert et al., 2020 [49]	12y/F	Germinoma	Yes	GTR	No	Tumor site (not otherwise described)	CBCDA + VP-16	-	No	Dense	No	18 months	-	18 months	Alive
Thong et al., 2020 [50]	12y/M	Germinoma	Yes	GTR	No	Radiation (not otherwise described)	Chemotherapy (not otherwise described)	-	No	Moderate	No	7 days	-	7 days	Alive
Tai et al., 2021 [51]	25y/M	Germinoma	Yes	GTR	No	Radiation (not otherwise described)	CDDP + VP-16	-	No	Moderate	No	8 months	-	8 months	Alive
Zhang et al., 2021 [52]	15y/M	Mature Teratoma	No	GTR	No	-	-	-	No	Sparse	No	12 months	-	12 months	Alive
Isaji et al., 2022 [53]	50y/F	Mixed tumor with a yolk sac tumor component	Yes	PR	No	Whole ventricular system 25.2Gy + Tumor site 32.4Gy		-	No	Moderate	No	18 months	-	18 months	Alive
Albina et al., 2022 [54]	33y/F	Germinoma	Yes	PR	No	Whole ventricular system 23.4Gy + Tumor site 45Gy	-	-	No	Moderate	No	6 months	-	6 months	Alive
Present case	24/F	Germinoma	Yes	STR	No	Whole ventricular system 24Gy + Tumor site 24Gy	CBCDA + VP-16	No abnormality	No	Moderate	No	5 months	-	5 months	Alive

A female predilection (male-to-female ratio: 1:1.3) was observed. When confined to pure germinoma, the female predilection was even more pronounced (1:1.9). The histology was pure germinoma in 26 cases (76%), with the other 4 cases harboring a germinoma component in mixed GCT (12%). The remaining 4 cases (12%) were diagnosed as teratoma. Up to 32 cases (94%) showed no evidence of hydrocephalus at presentation. Gross total resection, subtotal resection, and partial resection were performed in 12 (35%), 12 (35%), and 10 (30%) cases, respectively. Postoperatively, RT alone, CMT alone, and RT + CMT were administered in 9 cases (26%), 2 cases (6%), and 17 cases (50%) respectively. Six cases did not receive adjuvant treatments due to early death after surgery (Cases 1, 11, and 24), early publication before advancing to postoperative treatment (Case 17), or the decisions of the treatment team (Cases 22 and 31).

Among the 27 cases that underwent RT, the irradiated area was described in 21 cases. This included whole-ventricular irradiation (7 cases, 33%), whole-brain irradiation (5 cases, 24%), gamma-knife surgery (2 cases), radiation to posterior fossa and a boost to the tumor bed (1 case), and radiation to craniospinal axis plus a boost to the tumor bed (2 cases, 10%).

**Figure 4:**
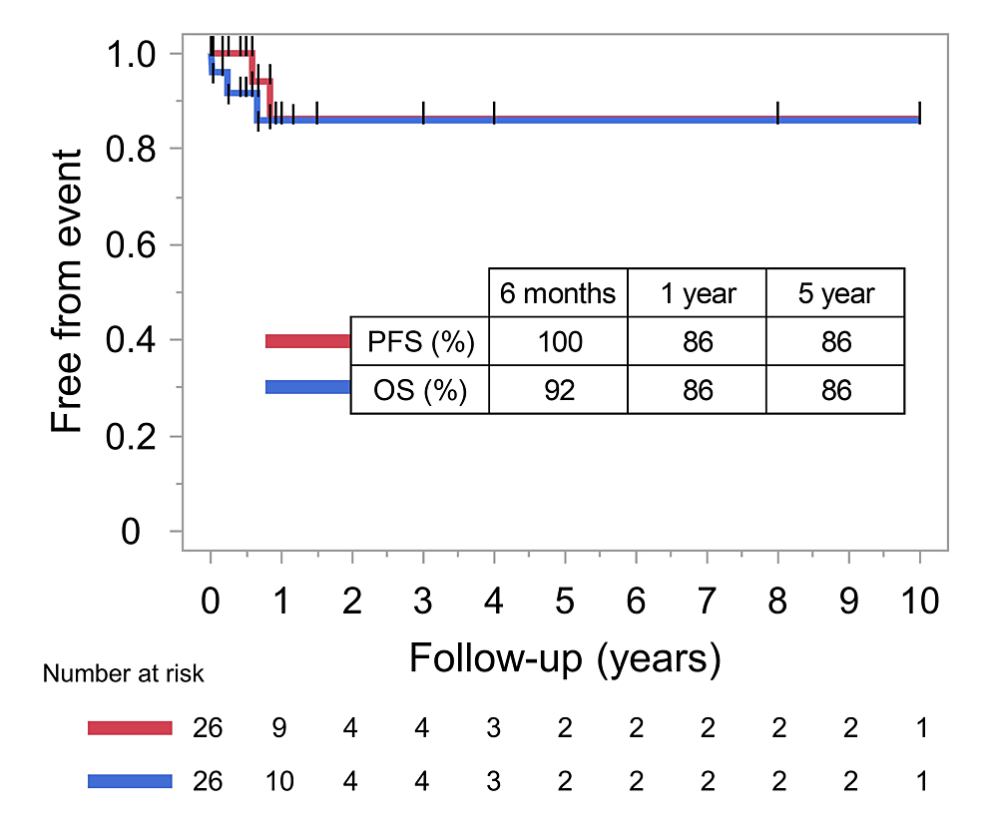
The PFS and OS of pure germinoma were calculated using the Kaplan-Meier method The 6-month, 1-year, and 5-year overall survival (OS) were 84%, 80%, and 64%, respectively. The 6-month, 1-year, and 5-year progression-free survival (PFS) were 96%, 87%, and 69%, respectively.

Case 17 was excluded from further analyses due to the lack of detailed follow-up data, leaving 33 cases for further analyses. Median follow-up was 11.0 months (7 days to 9 years; mean, 20.2 months). At the last follow-up, 27 patients (79%) remained alive. Six of seven mortality cases were because of cardiac and/or respiratory failures. A patient died from sudden cardiac arrest 12 days after the surgery, a patient died from pneumonia and gastrointestinal bleeding 18 days after the surgery, and four patients died from respiratory failure 3 to 41 months after the surgery. The remaining single case also exhibited respiratory failure, although the direct reason was postoperative CNS infection, which was a ventriculitis caused by methicillin-resistant *Staphylococcus aureus*. The 6-month, 1-year, and 5-year overall survival (OS) were 84%, 80%, and 64%, respectively. The 6-month, 1-year, and 5-year progression-free survival (PFS) were 96%, 87%, and 69%, respectively. Relapse was observed in 4 cases, and all of which were local recurrences. Two of the four recurrent cases were NGGCTs, including one immature teratoma and mixed GCTs with an embryonal carcinoma component. One recurrent case did not undergo RT, while the other three cases with recurrence underwent local RT at initial treatment. The radiation dose was described in two cases, with 40 Gy to the tumor site in one, 32.4 Gy to the tumor site, and 21.6 Gy to the posterior fossa in the other. Total radiation dose was described in 15 cases, and those cases with recurrence did not appear to receive a lower dose compared to cases without recurrence. When confined to cases of pure germinoma, the 6-month, 1-year, and 5-year OS rates were 92%, 86%, and 86%, respectively. Six-month, 1-year, and 5-year PFS rates were 100%, 86%, and 86%, respectively (Figure 4).

Clinicopathological characteristics and diagnosis

In general, primary CNS GCTs predominantly occur in juvenile and young adult males [8,11]. The majority of patients are teenagers. A clear male predilection is seen (male-to-female ratio: 1.9:1), except at neurohypophyseal locations (male-to-female ratio: 1:1) [11,15]. Our study revealed that the mean and median age for medulla oblongata GCT was 22 years, and less than half of the cases were teens (15 cases; 44%), showing a significantly older patient group than other GCTs at more common locations. Strikingly, a clear female predilection (1:1.3) was seen for medulla oblongata GCT, representing another noteworthy characteristic. These findings suggest that the typical patient profile of medulla oblongata GCT is a woman in her early 20s, matching our case, and deviating from the representative GCT patient profile of a male teenager, though the possibility of selection bias cannot be excluded.

Germinoma comprises 41-65% of histopathological diagnoses of primary CNS GCTs [11,55], and tumors with a germinoma component represent up to 75% of primary CNS GCTs [8]. In our study, pure germinoma was the predominant histological classification (76%), and 88% of cases included a germinoma component.

Another outstanding characteristic of this entity was the striking rarity of accompanying hydrocephalus (6%). We think the intraoperative findings of our illustrative case would be indicative of the underlying rationale. The tumor indeed arose from the medulla oblongata and projected toward the fourth ventricle. However, the tumor did not invade the floor of the fourth ventricle but instead invaded the roof of the fourth ventricle. The tumor extended through the tela choroidea and reached the inferior medullary velum, whereas the foramina of Magendie and Luschka were clearly spared. This appeared to avoid obstruction of the cerebrospinal fluid pathway. The case with evident hydrocephalus was due to obstruction of the cisterna magna at the level of the foramen magnum, not obstruction of the fourth ventricle [35]. We concur with the above characteristic pattern of tumor extension as one of the features of medullary GCT.

Outcomes and risk factors

The 5-year OS was 64%, and 86% when confined to pure germinoma. Of note, six of the seven deaths were due to cardiac and/or respiratory failure, supposedly related to the brainstem location near vital cardiac and respiratory centers. This is another outstanding and thought-provoking feature of this entity [47].

The 5-year PFS of GCT was 69%, and all relapses were local. No cases needed ventriculo-peritoneal shunt during the course. When confined to pure germinoma, 5-year PFS was 86%. This rate is comparable to germinomas in general, with 5-year PFS reported as 83.5-86.9% [14,56]. Atypical locations are known to predict worse prognosis [14], but this may not necessarily be true for medullary occurrence. No cases needed ventriculo-peritoneal shunt during the course. The observation of only local recurrence does not corroborate the use of extensive radiation fields such as whole-brain or spinal irradiation for medulla oblongata germinoma.

We tried to elucidate the factors that affect the OS of medullary germ cell tumors, which failed owing to the rarity and relatively short follow-up periods. Prognostic factors such as age, sex, RT, CMT, histology, the extent of resection, degree of lymphocyte infiltration, and presence of dissemination at presentation were included in the anlysis. Univariate analysis revealed that RT (P=0.023) predicts a better OS and pure germinoma (P=0.089) has a tendency for a better OS, though insignificant. The limited number of cases prevented us from advancing to multivariate analysis. In terms of CNS germ cell tumors, RT is expected to provide a potential for cure. Though partially effective, CMT is said to be less effective than RT. Accordingly, multimodality therapy is currently considered the best practice [8]. While we could not prove the same results regarding the medullary germ cell tumors, we believe that similar results would be observed with further accumulations of cases. Furthermore, the precise regimen of the CMT should be discussed in order to achieve better outcomes. 

Guarded interpretation of our data is reasonable, but a prospective study is warranted to answer the question of optimal treatment for medulla oblongata germinoma.

## Conclusions

We presented a case of germinoma of the medulla oblongata. Although rare, factors such as female sex, occurrence in the twenties, and a homogenously enhancing lesion without hydrocephalus should raise suspicion for this diagnosis. A review of the literature indicates the need for attention to cardiopulmonary issues as they are dominant causes of mortality, related to critical functions of this region. The local relapse pattern does not abrogate the possibility of skipping extensive radiation fields, although prospective studies are warranted. 
